# Sublethal vancomycin-induced ROS mediating antibiotic resistance in *Staphylococcus aureus*

**DOI:** 10.1042/BSR20140167

**Published:** 2015-12-24

**Authors:** Gui-qiu Li, Feng Quan, Ting Qu, Juan Lu, Shu-lan Chen, Lan-ying Cui, Da-wen Guo, Yong-chen Wang

**Affiliations:** * Department of Laboratory Diagnosis, the first Affiliated Hospital of Harbin Medical University, No. 194, Rd. Xuefu, District Nangang, Harbin, 150081, Heilongjiang Province, China; † Department of Rheumatology, the Second Affiliated Hospital of Harbin Medical University, Harbin, 150081, Heilongjiang Province, China; ‡ Department of Pharmacy, the first Affiliated Hospital of Harbin Medical University, No. 194, Rd. Xuefu, District Nangang, Harbin, 150081, Heilongjiang Province, China; § The first Affiliated Hospital of Harbin Medical University, No. 194, Rd. Xuefu, District Nangang, Harbin, 150081, Heilongjiang Province, China

**Keywords:** antibiotic resistance, heterogeneous vancomycin resistant *S. aureus* (hVRSA), reactive oxygen species, vancomycin

## Abstract

*Staphylococcus aureus* is the leading cause of many human infectious diseases. Besides infectious dangers, *S. aureus* is well-known for the quickly developed drug resistance. Although great efforts have been made, mechanisms underlying the antibiotic effects of *S. aureus* are still not well clarified. Recently, reports have shown that oxidative stress connects with bactericidal antibiotics [Dwyer et al. (2009) Curr. Opin. Microbiol. **12**, 482–489]. Based on this point, we demonstrate that reactive oxygen species (ROS) induced by sublethal vancomycin may be partly responsible for the antibiotic resistance in heterogeneous vancomycin resistant *S. aureus* (hVRSA). Sublethal vancomycin treatment may induce protective ROS productions in hVRSA, whereas reduction in ROS level in hVRSA strains may increase their vancomycin susceptibility. Moreover, low dose of ROS in VSSA (vancomycin susceptible *S. aureus*) strains may promote their survival under vancomycin conditions. Our findings reveal that modest ROS generation may be protective for vancomycin resistance in hVRSA. These results recover novel insights into the relationship between oxidative stress and bacterial resistance, which has important applications for further use of antibiotics and development of therapeutics strategies for hVRSA.

## Abstract (precis)

*S. aureus* may cause many human infectious diseases, which is well-known for the quickly developed drug resistance. Reports have shown that oxidative stress connects with bactericidal antibiotics. Our results exhibit that at least induced ROS may be beneficial to vancomycin resistance in two strains of hVRSA. The present findings help to recover novel insights into the relationships between oxidative stress and bacterial resistance, which has important implications for further use of antibiotics and development of therapeutics strategies for hVRSA.

## INTRODUCTION

*Staphylococcus aureus*, a Gram-positive cocci, is one of the most common causes of nosocomial infections, from skin and chronic bone infections to devastating septicaemia and endocarditis [1]. Besides infectious dangers, *S. aureus* is well-known for the ability to develop quick drug resistance [2]. The acquisition of anti-microbial resistance and changing patterns of *S. aureus* become common themes in staphylococcal literature [3]. For example, vast majority of current hospital-acquired *S. aureus* are resistant to usual antibiotics, such as penicillin and methicillin [4]. Although many new antibiotic drugs have come into use, *S. aureus* showed a unique ability to quickly response to each new challenge with the development of new resistance. Thus, it is of emergency to clarify mechanisms of drug resistance in *S. aureus.*

To treat *S. aureus* with penicillin and methicillin resistance, vancomycin, a glycopeptide antibiotic, was widely used [5,6]. During early times for vancomycin treatment, there was no indication that vancomycin resistance in *S. aureus* was likely to be a problem. However, since reports of reduced vancomycin susceptibility in clinical isolates of *S. aureus* decades ago, the heterogeneous vancomycin resistant *S. aureus* (hVRSA) has become a significant concern in medical community [7–9]. Generally, *S. aureus* resistance to antibiotics is mediated by various mechanisms [10]. It seems likely that the thickening of cell wall is closely associated with vancomycin resistance in hVRSA strains [11,12]. And, sequential point mutations in key global regulatory genes contribute to hVRSA phenotypes, which are connected with cell-wall thickening and restricted vancomycin access to its site of activity [13]. Also, it has been reported that horizontal gene transfer may contribute to resistance traits in *S. aureus* to methicillin and vancomycin [14]. And endogenous resistance, which is acquired through the process of random mutation and selection under antibiotic pressure, may play an important role in clinical resistance [15]. Nevertheless, although much progress has been made, how vancomycin resistance in *S. aureus* is precisely regulated needs further elucidation.

In recent years, reactive oxygen species (ROS) have been proposed to be highly correlated with the antibiotics and bacterial killing [16]. A hallmark paper showed that antibiotics induce ROS by activating the electron transport chain and kill bacteria by causing destabilization of cell structures [17]. On the other hand, ROS formation due to treatment with bactericidal antibiotics could lead to an increase in mutation rates, which can result in the emergence of multi-drug resistance [18]. Conversely, two recent reports declare that no correlation between an individual cell's probability of survival in the presence of antibiotic and its level of ROS, suggesting that ROS do not play a role in killing of bacterial pathogens by antibiotics [19,20]. Therefore, the relationship of antibiotic-induced ROS and bacterial killing is complicated and needs further clarification.

To investigate the mechanism of how hVRSA strains resists vancomycin and the role of ROS production in this process, we examined the survival of two vancomycin susceptible *S. aureus* (VSSA) and two hVRSA strains by gradient of vancomycin treatment. Interestingly, we found that hVRSA strains are not susceptible to sublethal vancomycin treatment, which was accompanied by ROS productions. Expectedly, the VSSA strains were susceptible to sublethal vancomycin treatment. And the induced-ROS level was much lower in VSSA than that in hVRSA strains. Moreover, we verified that additive treatment of low-dose hydrogen peroxide increases hVRSA survival under conditions with or without vancomycin, but overwhelmed oxidative stress kills hVRSA by high-dose hydrogen peroxide. Taken together, our results exhibit that at least induced ROS may be beneficial to vancomycin resistance in two strains of hVRSA. Our work recovers novel insights into the relationships between oxidative stress and bacterial resistance, which has important implications for further use of antibiotics and development of therapeutics strategies for hVRSA.

## MATERIALS AND METHODS

### Bacterial strains, growth conditions and viability assays

hVRSA and VSSA strains were obtained from the first Affiliated Hospital of Harbin Medical University (introduced from West China Hospital of Sichuan University) and their vancomycin resistance status was determined by the originating laboratory and confirmed by minimum inhibitory concentration (MIC) testing [21]. *N*-acetyl-cysteine (NAC) was purchased from Sigma–Aldrich. To determine the cell viability of hVRSA and VSSA strains, overnight cultures (16 h) were diluted by 100 times and grown aerobically in LB at 37°C to an *A*_600_ of 0.3. Then, different concentrations of vancomycin were added into cells. After different incubation times (specified in each figure), cells were taken and diluted in PBS buffer, spotted on LB agar and incubated at 37°C for 16 h. Cell survival was determined by counting colony-forming units (CFU) [22].

### Endogenous ROS production

As for the endogenous ROS quantifications, cells of hVRSA and VSSA strains were harvested, washed twice with PBS and then re-suspended. The probe 2′,7′-dichlorofluorescein diacetate (Sigma) was then added to cell suspensions, at a final concentration of 10 μM. Fluorescence intensity (FI) was acquired during 2 h using 96-well spectrofluorimeters (*E*_x_/*E*_m_=485/538 nm). The FI value was normalized in relationship to the *A*_600_ nm of each culture for quantifications [23].

### Quantitative real-time RT-PCR

For real-time reverse transcription-PCR experiments, 0.5 μg of hVRSA and VSSA total RNA were used to synthesize cDNA with the First Strand cDNA Synthesis Kit for RT-PCR from Roche Applied Science. Real-time PCR experiments were performed in a Bio-rad iQ5 instrument using SYBR Green I Kit according to the manufacturer's instructions (Roche Applied Science). The amplification reactions were carried out using previously synthesized cDNA as the initial template and each reaction mixture contained *sodA* (superoxidase dismutase A) primers (F-5′-AGGCCATTGGTCGTATTT-3′ and R-5′-GCAAATCA TCTAAGGGCTATG-3′). The expression ratio of the target gene was determined relatively to a reference gene, the 16S rRNA, whose transcription remains invariant under the tested conditions [23].

### Statistical analysis

Statistical analysis was carried out using ImageJ and GraphPad Prism softwares. Quantitative data were shown as x ± s using ANOVA tests for comparisons.

## RESULTS

### hVRSA strains resist to vancomycin-mediated bacterial killing

**Figure 1:**
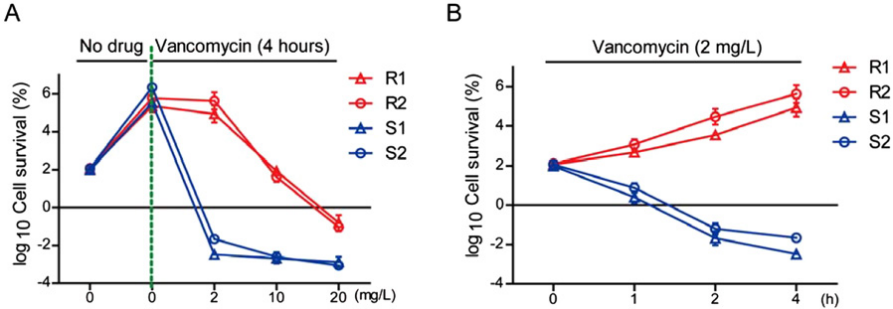
The killing effect of vancomycin on *Staphylococcus aureus* Cell survival of *S. aureus* was measured by CFU per millilitre and normalized relative to no vancomycin or time zero points (defined as 100). Results are averages of five independent experiments. Data represent mean ± S.E.M. (**A**) By vancomycin treatment (4 h) with gradient concentrations (~0–20 mg/l), R1 and R2 strains of hVRSA (red) resisted vancomycin at 2 mg/l group and developed cell death at ~10–20 mg/l groups. Whereas S1 and S2 strains of VSSA (blue) were quickly killed by both low and high vancomycin treatment. **(B)** By sublethal vancomycin treatment (2 mg/l), R1 and R2 strains of hVRSA (red) survived from 0 to 4 h. Whereas S1 and S2 strains of VSSA (blue) were gradually killed as time lapsed.

To compare the vancomycin susceptibility in hVRSA and VSSA strains, we examined the survival rate of *S. aureus* by different concentrations and times of vancomycin treatment. Gradient concentrations of vancomycin were added to the exponentially-growing culture of two hVRSA and VSSA strains and samples were taken for a colony count 4 h later. The results showed that hVRSA strains could resist to cell death induced by low concentrations of vancomycin (2 mg/l) and only high concentrations of vancomycin (10 or 20 mg/l) exhibited bacterial killing effect (Figure 1A). However, VSSA strains were more susceptible to vancomycin treatment, in either low or high concentrations of vancomycin (Figure 1A). To better study the killing effect of vancomycin on hVRSA and VSSA strains, we applied to test in a time-dependent experiment. The survival curves showed that low concentrations of vancomycin (2 mg/l) may effectively kill VSSA strains, but not kill hVRSA strains (Figure 1B). Thus, low concentrations of vancomycin may be verified to kill VSSA strains, but not kill hVRSA strains.

### Sublethal vancomycin treatment induces ROS production in hVRSA strains, not in VSSA strains

**Figure 2:**
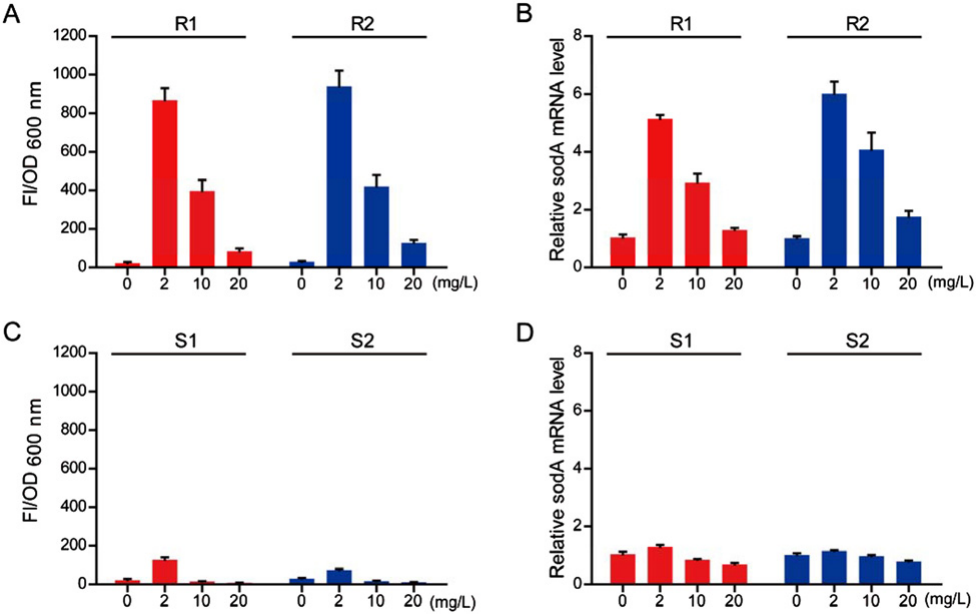
Sublethal vancomycin treatment induces ROS production in hVRSA, not in VSSA By fluorescence assays, it was found that endogenous ROS level is induced by vancomycin in hVRSA strains for 4 h (**A**), not in VSSA strains (**C**). Noted that the ROS production begins to decrease exposed to high concentrations of vancomycin treatment. Real-time PCR results showing that mRNA level of *sodA* (normalized to control) was consistently altered by vancomycin treatment in hVRSA strains (**B**) and not altered much in VSSA strains (**D**). Results are averages of five independent experiments. Data represent mean ± S.E.M.

Since low concentrations of vancomycin (2 mg/l) could not effectively kill hVRSA strains, we would like to study how these two hVRSA strains develop vancomycin resistance. It is noted that ROS formation may increase mutation rates and result in the emergence of multi-drug resistance in *E. coli* strains [18]. We then proposed that sublethal vancomycin treatment may induce ROS production in hVRSA strains and cause antibiotic resistance. Based on this hypothesis, we investigated the ROS production by vancomycin treatment in current hVRSA strains. Results showed that endogenous ROS level was dramatically increased by vancomycin treatment in hVRSA strains. Of note, we found that low concentrations of vancomycin (2 mg/l) may induce more ROS productions than that in higher concentrations (10 or 20 mg/l; Figure 2A). To confirm ROS productions in hVRSA strains, we further examined the expression of ROS related gene *sodA*. Results showed that transcriptional level of *sodA* is dramatically induced by vancomycin treatment in hVRSA strains and low concentrations of vancomycin induced higher *sodA* expression than that in high concentration counterparts (Figure 2B). Parallely, we found that ROS level was not dramatically induced by vancomycin treatment in two VSSA strains. Neither low (2 mg/l) nor high (10 or 20 mg/l) concentrations of vancomycin could induce dramatic ROS productions (Figure 2C). Similarly, the transcriptional level of *sodA* was also not induced by vancomycin treatment in VSSA strains (Figure 2D). Based on these findings, we assume that sublethal vancomycin may induce ROS production in two hVRSA strains, which may contribute to their vancomycin resistance.

### Reduction in ROS level in hVRSA strains may increase their vancomycin susceptibility

**Figure 3:**
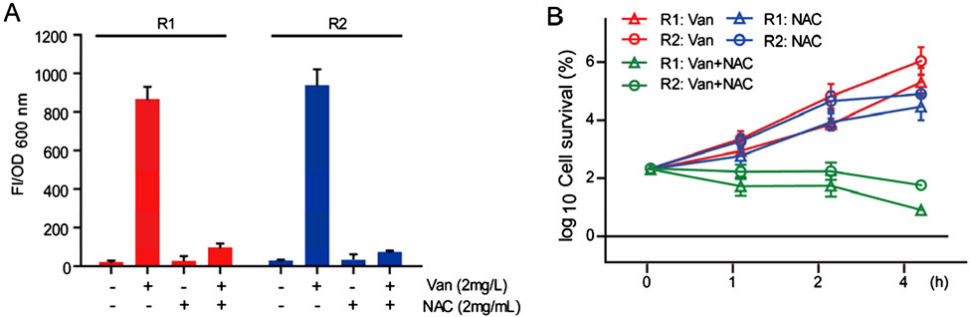
Reduction in ROS level in hVRSA strains may increase their vancomycin susceptibility By fluorescence assays, it was found that vancomycin-mediated (2 mg/l) ROS generation was blocked by NAC treatment (2 mg/ml; **A**). Further, parallel reduction in cell survival of hVRSA strains was noted by NAC treatment (2 mg/ml) in the presence of sublethal vancomycin (2 mg/l; **B**). We also noted that NAC alone treatment could induce modest killing effect compared with vancomycin alone groups. Results are averages of five independent experiments. Data represent mean ± S.E.M.

As for hVRSA strains develop ROS by vancomycin treatment, we wonder whether these ROS generation may protect hVRSA strains against vancomycin. To test this hypothesis, we applied anti-oxidants to vancomycin-treated hVRSA strains. NAC is a ROS scavenger and GSH precursor [24], which has anti-bacterial properties [25]. Therefore, we treated current hVRSA strains with vancomycin (2 mg/l) plus NAC (2 mg/ml) [25]. To confirm NAC's effect of ROS scavenging, we examined ROS levels by fluorescence assays. Results showed that NAC addition dramatically reduced endogenous ROS level in vancomycin-treated hVRSA strains (Figure 3A). Moreover, by counting CFU, we found that NAC addition may further reduce the survival of hVRSA strains under vancomycin conditions. Results showed that vancomycin plus NAC treatment could reduce the log_2_ cell survival by 57.04% (R1), 55.22% (R2) after 2 h and 85.40% (R1), 72.63% (R2) after 4 h compared with their vancomycin-treated counterparts (Figure 3B). We noted that NAC treatment alone reduced cell survival after 4 h in two hVRSA strains (Figure 3B). Specifically, the values of log_2_ cell survival of NAC groups reduced by 16.46% (R1) and 19.30% (R2) compared with their vancomycin-treated counterparts in 4 h. Whereas in 2-h groups, the values of log_2_ cell survival of NAC groups was not statistically different from vancomycin counterparts. We attribute this modest killing (4-h group) to NAC anti-bacterial properties. After all, NAC may act as a non-antibiotic drug that inhibits *Staphylococcus* growth in ~4–8 mg/ml concentrations [25]. Nevertheless, all these results indicate that reduction in ROS level in hVRSA strains may indeed increase their vancomycin susceptibility.

### Low dose of ROS in VSSA strains may promote cell survival under vancomycin conditions

**Figure 4:**
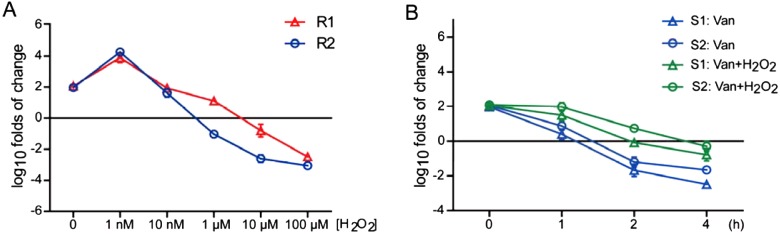
Low dose of ROS in VSSA strains may promote cell survival under vancomycin conditions (**A**) Additive ROS stress with vancomycin reduces hVRSA survival. The survival curve showing that extra oxidative stress by hydrogen peroxide increases cell survive by low-dose treatment (1 nM) and induces cell death as doses increases (1–100 μM). (**B**) The survival curve showing that extra hydrogen peroxide treatment (1 nM) in the presence of vancomycin (2 mg/l) may improve cell survival of VSSA strains. Results are averages of five independent experiments. Data represent mean ± S.E.M.

The consistent increased ROS productions and hVRSA survival under vancomycin conditions indicate that sublethal vancomycin may induce endogenous ROS to protect from cell death. To confirm this finding, we applied to increase ROS level by extra hydrogen peroxide treatment and examined the survival of these two hVRSA strains. The results showed that low-dose treatment (1 nM) of hydrogen peroxide increased the survival of hVRSA strains, whereas high-dose treatment of hydrogen peroxide killed the hVRSA strains (Figure 4A). So at least proper ROS level may be beneficial to the survival of hVRSA strains. Further, we applied low dose of hydrogen peroxide to VSSA strains under vancomycin conditions. The survival curve showed that low-dose treatment (1 nM) of hydrogen peroxide may partly improve cell survival by vancomycin treatment. As time lapsed, this protective effect was gradually diluted, which might be caused by the invalidated hydrogen peroxide (Figure 4B). Nevertheless, our work indicate that proper ROS level may contribute to the antibiotic resistance for *S. aureus* strains.

## DISCUSSION

**Figure 5:**
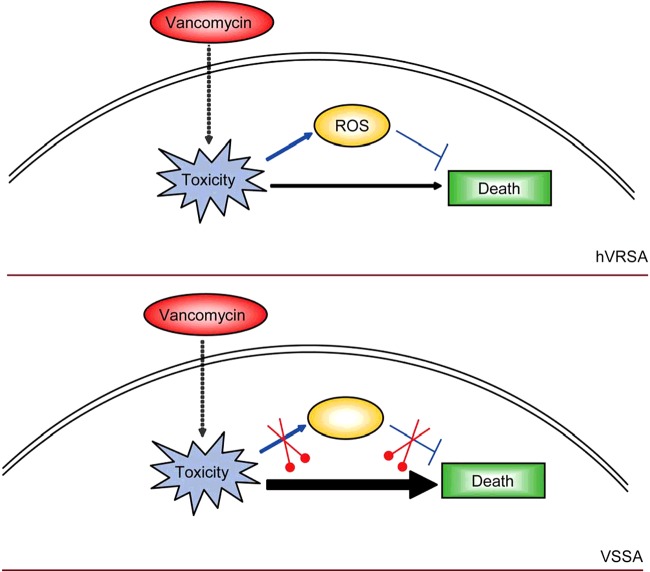
Model highlighting the relationship of endogenous ROS production and antibiotic resistance in hVRSA Schematic representation highlighting the relationship of endogenous ROS production and antibiotic resistance in hVRSA. In hVRSA strains, vancomycin treatment induces cell toxicity and ROS productions, which may help to resist cell death within a certain range. Whereas in VSSA strains, vancomycin directly induces cell toxicity and cell death, which may be partly due to the failed protective ROS productions.

Clinical situations where bacteria (e.g. *S. aureus*) resist to antibiotics can occur with terrible infections. *S. aureus* is a human pathogen with antibiotic resistance, which is responsible for most wound and hospital-acquired infections [26]. Although broad attention has been brought to the study of mechanisms of antibiotic resistance in *S. aureus* infectious diseases, it's still largely unknown about the antibiotic-resistant mechanism of *S. aureus.* In the present study, we establish a molecular mechanism whereby sublethal vancomycin may induce protective ROS productions in hVRSA. Whereas higher concentrations of vancomycin exhibit killing effect accompanied by reduced protective ROS level (Figure 5). Our work will help to uncover novel relationships between ROS and bacterial antibiotic resistance and provide new insights to the prevention of clinical *S. aureus* infections.

The relationship between ROS and antibiotic killing is complicated. In decades, most clinical antibiotics target cell-wall assembly, protein synthesis or DNA replication [19]. Recent reports have raised the point that although antibiotics block growth by directly inhibiting the targets mentioned above, they may owe their lethal effects to the indirect creation of ROS to impair bacterial DNA [19]. Indeed, antibiotics could induce ROS production by activating electron transport chain, which kills bacterial cells [17]. However, this traditional view was greatly challenged by that no correlation between an individual cell's probability of survival in the presence of antibiotic and ROS [19,20]. Moreover, antibiotics could even induce mutagenesis by stimulating the production of ROS.

Although oxidative stress has been implicated as one of the mechanisms whereby bactericidal antibiotics kill bacteria, the formation of ROS may play distinct roles in bacteria killing and survival. In the present study, we provide new insights that induced-ROS may be even beneficial to the survival of hVRSA strains under vancomycin conditions. It is of note that only low-concentrations of ROS may be helpful for the antibiotic resistance, which is induced by sublethal vancomycin. Either high concentration of vancomycin or additive oxidative stress may impair protective mechanism of hVRSA strains to antibiotics. On the other hand, we notice that when treated by vancomycin, VSSA strains seem to fail to induce ROS, which may lead to the incomplete bacterial protective systems. This result is consistent with previous studies, indicating that antibiotics may not appear to always cause oxidative stress in bacteria [27] and the killing effect by bactericidal antibiotics does not always depend on ROS [19,20].

In conclusion, our findings verify that induced ROS may be protective for vancomycin treatment in two strains of hVRSA. It recovers novel insights into the relationships between oxidative stress and bacterial resistance, which has important implications for further use of antibiotics and development of therapeutics strategies for hVRSA.

## Abbreviations

CFU: colony-forming units
FI: fluorescence intensity
hVRSA: heterogeneous vancomycin resistant *S. aureus*
MIC: minimum inhibitory concentration
NAC: *N*-acetyl-cysteine
ROS: reactive oxygen species
VSSA: vancomycin susceptible *S. aureus*
